# Hypoesthesia of midface by isolated Haller's cell mucocele^☆^

**DOI:** 10.1016/j.bjorl.2016.06.009

**Published:** 2016-07-22

**Authors:** Jeong Hwan Choi

**Affiliations:** Inje University, College of Medicine, Sanggye Paik Hospital, Seoul, South Korea

## Introduction

Anatomic variations in the nose and paranasal sinuses (PNS) do not necessarily indicate a pathologic state but can predispose some patients to sinus disease by causing obstruction that can lead to inflammatory disease. Migrating anterior or posterior ethmoidal air cells that pneumatize the roof of the maxillary sinus or floor of the orbit are also termed “infraorbital ethmoidal”, “orbitoethmoidal”, “maxilloethmoidal”, or “Haller's cells” (HCs) named after the Swedish anatomist Albrecht von Haller. The incidence of HCs reported by different researchers covers a wide range (2–45%).^1^

The variable sizes and location of HCs on the roof of the maxillary sinus adjacent to and above the maxillary sinus ostium, forming the inferior orbital wall and the lateral border of the infundibulum implies that they are in a key position to cause the constriction of the infundibulum and obstruction of the ostium associated with recurrent acute rhinosinusitis.

A study^2^ compared the influence of sinonasal anatomic variants, such as septal deviation, concha bullosa and HCs in the recurrence of acute rhinosinusitis. The presence of HCs and smaller infundibular widths were statistically associated with recurrence.

Haller's cells are usually seen in the floor of the orbit and roof of the maxillary sinus adjacent to and above the natural ostium of maxillary sinus. Although a Haller's cell is considered a normal anatomical variant, when enlarged it can significantly constrict the posterior aspect of the ethmoidal infundibulum and maxillary ostium from above. If such a cell becomes diseased, the natural ostium of the maxillary sinus may rapidly become obstructed, and secondary maxillary sinusitis may develop.

Haller's cells also can reach the infraorbital nerve.

Isolated infection of the Haller's cell is usually very rare and should be suspected in patients with facial pain and hypoesthesia. The diagnosis of a Haller's cell may be difficult on endoscopy due to its location and can only be identified on radiology. Herein is a description of a rare case of hypoesthesia of the cheek caused by isolated mucocele formed by a Haller's cell. To the best of my knowledge, there has been no report in the English literature of midface hypoesthesia due to isolated mucocele from a Haller's cell; a search was made in PubMed using search terms such as “Haller's cell” and “infraorbital ethmoid cell” without results. This study was approved by the institutional review board (n° 2015-12-010).

## Case report

**Figure 1:**
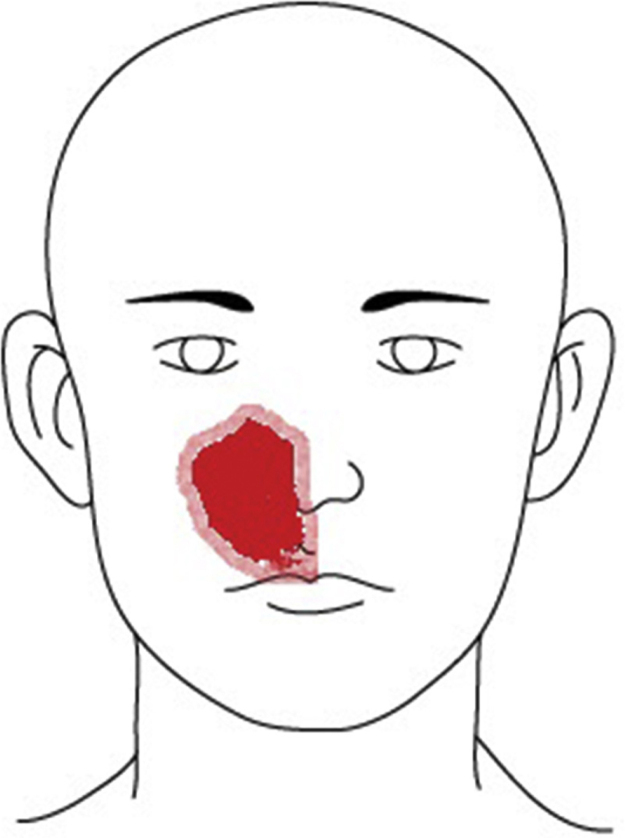
The patient presented to the neurology clinic of our hospital with complaints of right-side medial facial hypoesthesia (red shadow) for 3 days prior to arrival. This lesion corresponded to the dermatome of the infraorbital nerve.

A 49-year-old male patient presented to the neurology clinic of our hospital with chief complaint of right-side medial facial hypoesthesia for 3 days prior to arrival (Fig. 1). He had no other complaints of facial pain or nasal discomfort. Complete neurologic examination revealed no motor weakness or sensory change of body. MR imaging of brain showed nasal sinus inflammation, otherwise no abnormal signal intensity or mass lesion of the brain parenchyma or dura. He was referred to ENT clinic for further work up. His complaint was at the site of the infraorbital nerve dermatome. Nasal endoscopy with a 0°, 4 mm telescope revealed deviation of the nasal septum toward the right side and a widely patent middle meatus on the right. His symptoms did not change despite one week of antibiotics and mucolytics.

**Figure 2:**
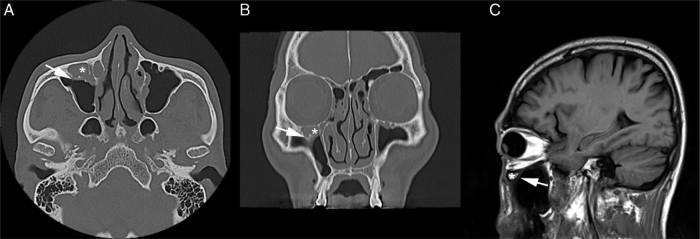
PNS CT revealed the presence of a soft tissue density in the right Haller's cell (*). The lesion abutted the infraorbital foramen (arrow) without definite evidence of communication. A, axial section; B, coronal section; C, sagittal section.

**Figure 3:**
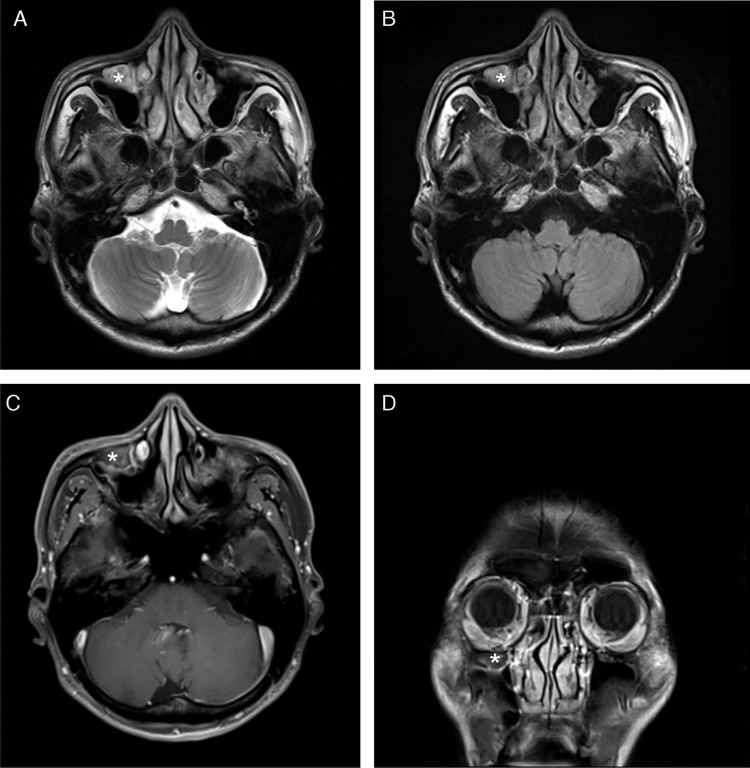
Brain MRI showed a fluid-filled Haller's cell (*) in the roof of the right maxillary sinus that was hyperintense on T2 axial image (A) and T2 FLAIR image (B). It was isointense on T1-weighted axial image (C) with subtle postcontrast enhancement (D).

Computed tomography (CT) scan of the PNS revealed the presence of a soft tissue density in the right Haller's cell. The lesion abutted the infraorbital foramen (Fig. 2A–C). MRI was thoroughly reviewed and showed a fluid-filled Haller's cell in the roof of the right maxillary sinus that was hyperintense on T2 and isointense on T1 weighted images, with subtle postcontrast enhancement (Fig. 3A–D).

The patient underwent right endoscopic sinus surgery with right mini Caldwell-Luc (CL) procedure in order to gain access to the opacified Haller's cell. Incision was made at the gingivobuccal sulcus from the root of the second molar tooth to the root of the canine fossa. The mucoperiosteal flap was elevated. A 30° nasal endoscope was passed through the mini-CL opening, and the Haller's cell was visualized. The Haller's cell was resected and was found to contain thick, purulent mucus. The incision was closed primarily after antral packing placement, and compression dressing was done. One week postsurgery, the patient's facial sense except for the right upper lip area was improved. Three weeks postsurgery, no hypoesthesia remained. The patient has stayed symptom free for 3 months.

## Discussion

HCs are the ethmoidal cells that develop into the floor of orbit and the roof of the maxillary sinus adjacent to and above the maxillary sinus ostium. These cells, if enlarged, can significantly constrict the posterior aspect of the ethmoidal infundibulum and superior aspect of the ostium of the maxillary sinus. Since the definition was not clear, a proposal was made in 1995 to give the name, “Haller's cell” to any “infraorbital cell of the anterior or posterior ethmoid, regardless of its origin”.^3^ It is a clinically significant anatomic variation, because it is a possible etiologic factor in recurrent maxillary sinusitis due to the cell's negative effect on maxillary sinus ventilation by narrowing the infundibulum and ostium.^2^ Such anatomical limitations may result in persistent or recurrent rhinosinusitis and headache. A case study reported headache attributed to the presence of HCs.^4^ It is difficult to identify any signs of disease in a patient with HCs. Diagnosis of Haller's cells is typically made by CT scan, as they cannot be identified by diagnostic nasal endoscopy because of their typical location lateral to the infundibulum. Easily seen on coronal PNS CT, they have been described as well-defined, round, oval, or teardrop-shaped, unilocular or multilocular radiolucencies with smooth borders that may or may not appear corticated, located medial to the infraorbital foramen.^5^ Asymptomatic in a majority of patients, Haller's cells may present with various symptoms. A mucocele of a Haller's cell can expand slowly, erode the roof of the maxillary sinus and extend into the orbital cavity. Expansion of the mucocele arising from a posteriorly located Haller's cell, when invading the orbit, can cause ophthalmological symptoms such as orbital edema, proptosis, diplopia, ptosis, visual or oculomotor disturbances, and pain in the eye.^6^

Inflammation of the Haller's cell is common in ethmoidal and maxillary sinus infection, but an isolated mucocele of this cell is very uncommon.^7^ A differential diagnosis of neuroma of the infraorbital nerve, cavernous hemangioma of the infraorbital canal, or mucocele of the septated compartment of the maxillary sinus must be on the differential. They are usually located in the roof of the maxillary sinus, in contrast to extra-antral mucoceles, which are usually found to arise from the floor of the sinus and push the floor of the antrum superiorly.^8^

There are no published reports describing the symptoms of hypoesthesia by isolated mucocele from a Haller's cell. Particularly in the absence of extensive associated mucosal changes, this condition may be easily overlooked unless specifically sought. The presence of HCs on coronal PNS CT in a patient with corresponding symptoms deserves consideration as the potential cause of these symptoms.

The infraorbital nerve, a direct extension of the maxillary division of the trigeminal nerve, passes along the floor of the orbit in the infraorbital groove and eventually exits the orbit through the infraorbital foramen and provides cutaneous innervation to the lower eyelid, the side of the nose, and the upper lip. The superior alveolar branch of the infraorbital nerve provides sensory innervation to the upper incisor, canine, and associated gingiva. This patient's area of hypoesthesia corresponded to the innervated infraorbital area (Fig. 1). Descension of the infraorbital nerve within the maxillary sinus is not an uncommon finding or not so rare, and is more prevalent in the setting of an ipsilateral infraorbital ethmoid cell.

The mucocele of the Haller's cell that invades the infraorbital area and clinically presents with signs of facial hypoesthesia is a rare entity and responds poorly to conventional medical treatment. In such a case, surgical intervention through a transnasal endoscopic approach is usually undertaken, but visualization of the cell may be difficult and access to the Haller's cell may require a mini CL approach, as in this case.

The management of pathologic Haller's cell is usually approached endoscopically through the middle meatus using a microdebrider to remove the uncinate process, including its inferior attachment. The cell is visualized and carefully uncapped with a curved microdebrider blade, and its inferior and medial portions are then carefully removed. The procedure widens the infundibulum and the outflow tract of the maxillary sinus. The superior portion of the Haller's cell is not dissected so that integrity of the orbital floor is not disturbed.^9^

## Conclusion

This is the first known report of facial hypoesthesia caused by Haller's cell mucocele. It was successfully resolved by endoscopic sinus surgery (ESS) combined with a mini-CL approach.

HCs have been related to different disease processes and symptoms, including recurrent or chronic sinusitis and sinogenic headaches without significant findings on physical examination. When medical therapy is ineffective, surgical therapy such as ESS should be considered in such cases.

## Conflicts of interest

The author declares no conflicts of interest.
